# Effects of a Novel Contextual Just-In-Time Mobile App Intervention (LowSalt4Life) on Sodium Intake in Adults With Hypertension: Pilot Randomized Controlled Trial

**DOI:** 10.2196/16696

**Published:** 2020-08-10

**Authors:** Michael P Dorsch, Maria L Cornellier, Armella D Poggi, Feriha Bilgen, Peiyu Chen, Cindy Wu, Lawrence C An, Scott L Hummel

**Affiliations:** 1 University of Michigan College of Pharmacy Ann Arbor, MI United States; 2 University of Michigan Health System Ann Arbor, MI United States; 3 University of Michigan Medical School Ann Arbor, MI United States; 4 Ann Arbor Veterans Affairs Health System Ann Arbor, MI United States

**Keywords:** hypertension, sodium intake, geofencing, mHealth

## Abstract

**Background:**

High dietary sodium intake is a significant public health problem in the United States. High sodium consumption is associated with high blood pressure and high risk of cardiovascular disease.

**Objective:**

The aim of this study was to evaluate the effect of a just-in-time adaptive mobile app intervention, namely, LowSalt4Life, on reducing sodium intake in adults with hypertension.

**Methods:**

In this study, 50 participants aged ≥18 years who were under treatment for hypertension were randomized (1:1, stratified by gender) into 2 groups, namely, the App group (LowSalt4Life intervention) and the No App group (usual dietary advice) in a single-center, prospective, open-label randomized controlled trial for 8 weeks. The primary endpoint was the change in the 24-hour urinary sodium excretion estimated from spot urine by using the Kawasaki equation, which was analyzed using unpaired two-sided *t* tests. Secondary outcomes included the change in the sodium intake measured by the food frequency questionnaire (FFQ), the 24-hour urinary sodium excretion, blood pressure levels, and the self-reported confidence in following a low-sodium diet.

**Results:**

From baseline to week 8, there was a significant reduction in the Kawasaki-estimated 24-hour urinary sodium excretion calculated from spot urine in the App group compared to that in the No App group (–462 [SD 1220] mg vs 381 [SD 1460] mg, respectively; *P*=.03). The change in the 24-hour urinary sodium excretion was –637 (SD 1524) mg in the App group and –322 (SD 1485) mg in the No App group (*P*=.47). The changes in the estimated sodium intake as measured by 24-hour dietary recall and by FFQ in the App group were –1537 (SD 2693) mg and –1553 (SD 1764) mg while those in the No App group were –233 (SD 2150) mg and –515 (SD 1081) mg, respectively (*P*=.07 and *P*=.01, respectively). The systolic blood pressure change from baseline to week 8 in the App group was –7.5 mmHg while that in the No App group was –0.7 mmHg (*P*=.12), but the self-confidence in following a low-sodium diet was not significantly different between the 2 groups.

**Conclusions:**

This study shows that a contextual just-in-time mobile app intervention resulted in a greater reduction in the dietary sodium intake in adults with hypertension than that in the control group over a 8-week period, as measured by the estimated 24-hour urinary sodium excretion from spot urine and FFQ. The intervention group did not show a significant difference from the control group in the self-confidence in following a low sodium diet and in the 24-hour urinary sodium excretion or dietary intake of sodium as measured by the 24-hour dietary recall. A larger clinical trial is warranted to further elucidate the effects of the LowSalt4Life intervention on sodium intake and blood pressure levels in adults with hypertension.

**Trial Registration:**

ClinicalTrials.gov NCT03099343; https://clinicaltrials.gov/ct2/show/NCT03099343

**International Registered Report Identifier (IRRID):**

RR2-10.2196/11282

## Introduction

High sodium intake is a significant public health problem in the United States [1]. The current federal guidelines advocate a daily sodium intake of less than 2300 mg/day [2]. However, the average sodium intake for Americans is approximately 3460 mg/day [3]. In a meta-analysis of 177,025 patients, higher sodium intake was associated with higher risk of stroke (relative risk 1.23, 95% CI 1.06-1.43) and a trend toward higher risk of cardiovascular diseases (relative risk 1.14, 95% CI 0.99-1.32) [4]. Interventions that lower sodium intake can decrease blood pressure levels and cardiovascular outcomes [5-8]. Implementing these interventions can be complex as it involves food manufacturers, restaurant chains, and community organizations.

Smartphones and mobile apps offer a scalable and pervasive opportunity to provide an intervention to individuals who frequently eat at restaurants or shop for high-sodium foods at grocery stores. Over half of the Americans consume 1-3 restaurant meals per week and 23% of the Americans consume 4 restaurant meals per week [9,10]. People who eat at restaurants frequently and buy high-sodium foods at grocery stores are the prime targets for this intervention, as about 77% of the sodium intake in the average American diet originates from processed and restaurant foods. We have developed a mobile app named as LowSalt4Life, which is an intervention aimed at reducing the sodium intake. Using smartphone sensors, the mobile app can detect when the patient arrives home or enters a restaurant or a grocery store and the restaurant or the grocery store that the patient has entered. This app provides a highly adaptive intervention wherein tailored messages are provided exactly when the patient needs help, also known as “just-in-time,” and with the precise information needed for that location, also known as “contextual.” Just-in-time adaptive interventions have the potential to dramatically improve patient behaviors in a variety of clinical settings [11].

The purpose of this pilot study was to evaluate the effectiveness of this mobile app intervention in providing contextual just-in-time adaptive push messages on dietary sodium intake and in improving the patient’s confidence in following a low-sodium diet.

## Methods

### Trial Design

The method of this trial has been published previously [12]. This clinical trial was a single-center, prospective, open-label randomized controlled trial that was conducted from June 2017 to March 2019. Participant recruitment was performed at Michigan Medicine, formerly the University of Michigan Health System, through the university recruitment platform and by sending letters to over 7000 patients who met our study criteria. Participants were randomized into the mobile app group (App group) or usual care group (No App group) in a 1:1 manner and stratified by gender using the University of Michigan Consulting for Statistics, Computing, and Analytics Research randomization instrument. Participants randomized to the App group received a 30-minute in-person training session and were educated to use the mobile app for 8 weeks by the study coordinator. The training session included information on how to use the mobile app. Participants randomized to the No App group received the standard of care at the University of Michigan. The University of Michigan guideline for hypertension management recommends modification of dietary sodium intake to less than 2400 mg/day. This study was approved by the University of Michigan Medical School Institutional Review Board (approval received on April 4, 2017), and all the participants provided written informed consent. This trial was listed at ClinicalTrials.gov (NCT03099343, received March 28, 2017) and was sponsored by the Agency for Healthcare Research and Quality (R21 HS024567).

### Participants in This Study

Patients older than 18 years diagnosed with hypertension, on antihypertensive therapy for at least 3 months, and using an iPhone were included. Patients were excluded if they had chronic kidney disease (CKD), heart failure, systolic blood pressure >180 mmHg, diastolic blood pressure >110 mmHg, insulin-requiring diabetes mellitus, or were taking loop diuretics, corticosteroids, or nonsteroidal anti-inflammatory medications. CKD was defined as known kidney damage (structural or functional abnormalities) or estimated glomerular filtration rate less than 60 ml/min/1.73 m^2^ (CKD stage 3, 4, or 5). The participants who provided the initial consent completed the Block Food Frequency Questionnaire (FFQ, NutritionQuest Inc) [13], and those with estimated baseline dietary sodium intake of less than 2300 mg/day were excluded before randomization. On October 1, 2018, the estimated dietary sodium intake for exclusion was changed to less than 2000 mg/day, with patients consuming 2000-2300 mg/day allowed to participate if the estimated sodium to kilocalorie ratio was greater than 1, as estimated by the FFQ. This change led to 4 patients being recruited that did not meet our initial study inclusion criteria. The 110-item Block FFQ was electronically self-administered and it recorded commonly consumed foods to estimate the nutrient and energy intake.

### Intervention

**Figure 1:**
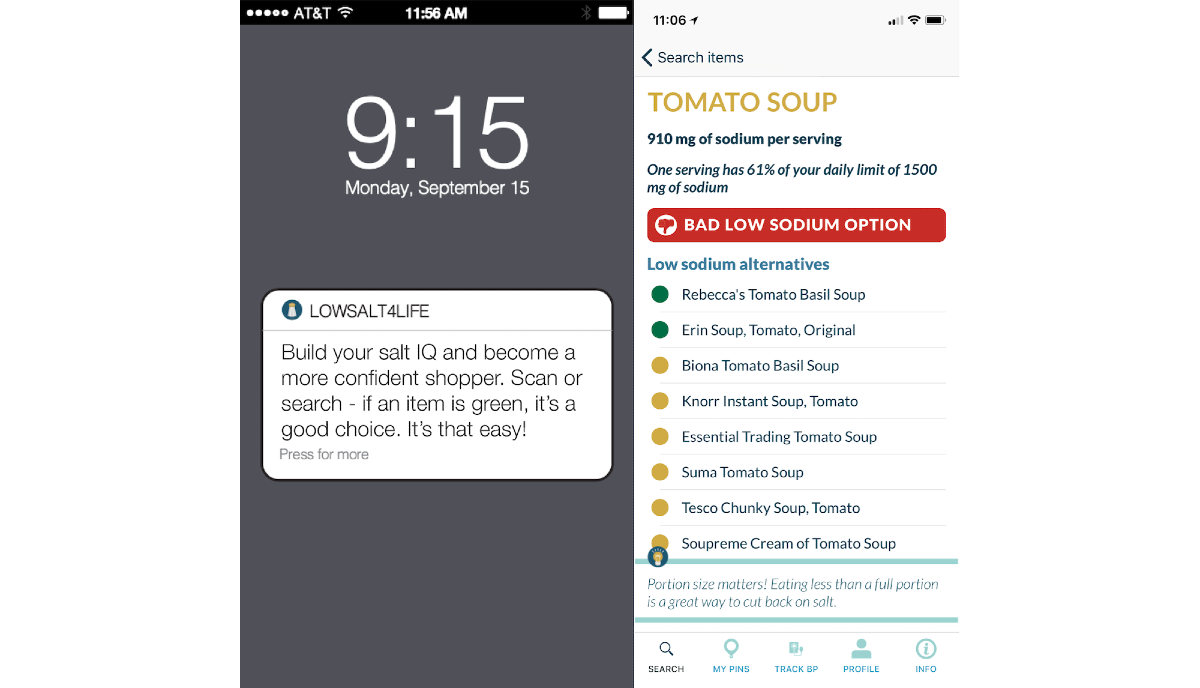
Example of a LowSalt4Life push notification and product search in the app.

The mobile app intervention method has been published previously [12]. Briefly, the intervention began with a baseline assessment of foods containing high sodium levels by using the Block Sodium Screener (NutritionQuest Inc). Participants selected alternatives to their 5 high-sodium foods and geotagged the places in which these foods were consumed or purchased (home, restaurant, or grocery store). In addition to these geolocations, a cloud-based web service was used to predict when the participant was entering a grocery store, restaurant, or home. Contextual just-in-time adaptive messages were provided to promote behavior change when a participant entered a grocery store, restaurant, or home. These messages were push notifications, which were tailored to the user’s confidence in following a low-sodium diet and linked to content in the mobile app. The mobile app showed the participant-selected alternatives to their 5 high-sodium foods, curated a list of low-sodium meal options at restaurants, provided users the capability to search restaurant menus that were prioritized by low sodium contents and/or the ability to scan the universal product codes of the grocery store items to find similar food options containing lower sodium contents. A standard nutrition database (Nutritionix) was used to provide the nutrition information at the grocery stores and restaurants through an application programming interface (API). Figure 1 shows an example of a push notification and product search in the app.

### Outcomes

**Figure 2:**
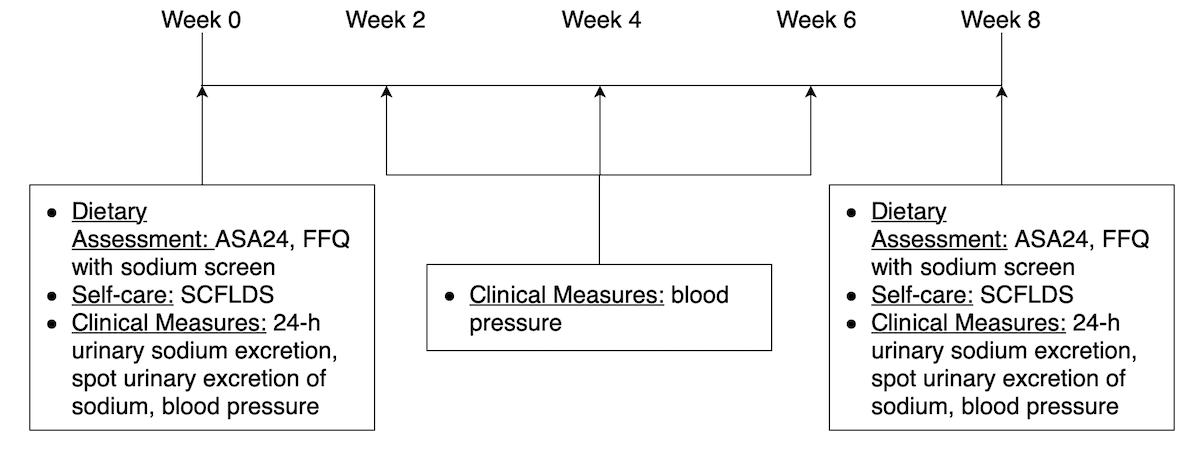
Study timeline and outcomes. ASA24: Automated Self-Administered 24-hour dietary food recall; FFQ: food frequency questionnaire; SCFLDS: Self-care Confidence in Following a Low-sodium Diet Scale.

The change in the dietary sodium intake was measured by both subjective and objective measures. Twenty-four-hour dietary recall data were collected and analyzed using the Automated Self-Administered 24-hour (ASA24) dietary assessment tool, version 2016, developed by the National Cancer Institute, Bethesda [14]. The ASA24 is an electronic 24-hour recall website that allows participants to self-administer the survey in a user-friendly manner. In addition to the Block FFQ 2014, a sodium screener survey was performed. This screener generates a score from the quantity and the frequency of consumption of high-sodium foods in the participant reports. The ASA24, FFQ, and sodium screener were administered at baseline and in week 8 of the study. Figure 2 shows the details of the timeline and all the outcomes.

The urinary excretion of sodium was measured by the 24-hour urinary collection method for monitoring the sodium intake. Participants were instructed to collect all urine voids for 24 hours and return them for analysis for sodium excretion by using the standard University of Michigan laboratory processes. The method of collection from the participants was written in our study procedures and reviewed with the participants prior to the baseline in-person visit. Participants were mailed the supplies, a collection worksheet, and an instruction letter for collecting the 24-hour urine samples. The study coordinator reviewed the collection sheet and the 24-hour urine samples with the participants during the 2 in-person visits. The Knuimann strategy was used to assess the completeness of the 24-hour urine collection, which uses urinary creatinine values and body weight [15]. Twenty-four-hour urine excretion was collected at baseline and after 8 weeks. In addition, the estimated 24-hour urinary sodium excretion was accomplished with a morning spot urine excretion of sodium at baseline and after 8 weeks. The Kawasaki formula was used to estimate the 24-hour sodium urinary excretion from a fasting morning urine sample [16]. The estimated 24-hour urine excretion of sodium calculated from spot urine was the primary endpoint because 24-hour urine collection can be difficult and inconvenient for participants, thereby leading to a lack of data and decreased statistical power. All urine sodium measurements were done on the same day.

The 7-item Self-care Confidence in Following a Low-sodium Diet Scale (SCFLDS) was used to quantify the participant’s confidence in following a low-sodium diet. It evaluates the patient’s confidence in the ability to select and prepare foods with low sodium contents [17]. The SCFLDS was given at baseline and week 8 of the study.

Blood pressure was measured on a biweekly basis by the participant. Participants were trained to take 3 blood pressure readings at each time point by using the standard American Heart Association guideline–based recommendations on how to measure blood pressure at home [18]. An average of 3 measurements was used as the participants’ blood pressure for that time point. Participants used their own automated blood pressure monitor at home for the study.

Mobile app data were collected to determine the extent to which the mobile app was used. The number of push notifications received by each participant and the use of the search functions for nutrition information was collected through the Nutritionix API. The Nutritionix API data is in aggregate and is not patient-specific. “Autocomplete” represents a search when typing in the app. “Universal Product Code Lookup” is the scanning function in the app. “Hits” represents the overall number of patient interactions with the nutrition information. Participants in the App group were also asked to complete a survey about their experience using the mobile app.

### Statistical Analysis

Baseline demographics were compared using a two-sided *t* test or chi-square with Fisher’s exact test, where appropriate, with the resulting *P* values for the tests. The primary endpoint was the estimated 24-hour urinary excretion of sodium from spot urine, which was estimated using the Kawasaki equation. Based on the data in the patients with hypertension, we expected the 24-hour urinary excretion of sodium to decline from 3400 (SD 1200) mg/day to 2400 (SD 1200) mg/day in the App group and a decline from 3400 (SD 1200) mg/day to 3300 (SD 1200) mg/day in the No App group [6]. A sample size of 24 patients (12 in each group) and 32 patients (16 in each group) would offer 80% and 90% power, respectively, for a 35% reduction in sodium intake. To account for patient dropout or incomplete follow-up, a total sample of 50 patients was identified as the study target enrollment. A two-sided unpaired *t* test was used to compare the change in each measure of sodium intake over time in the App versus No App groups.

Although not powered to determine the impact, other measures important for reducing sodium intake were evaluated. The Wilcoxon rank-sum test was used for continuous measures that were not distributed normally. The change in the self-confidence in following a low-sodium diet was analyzed using the two-sided *t* test. Repeated measures analysis of variance was used to determine the change in the blood pressure over time in each group. *P* values less than .05 were considered statistically significant. Data were represented as mean (SD) or number (%), unless otherwise noted. All analyses were performed using SAS software (version 9.4, SAS Institute, IBM Corp).

## Results

**Figure 3:**
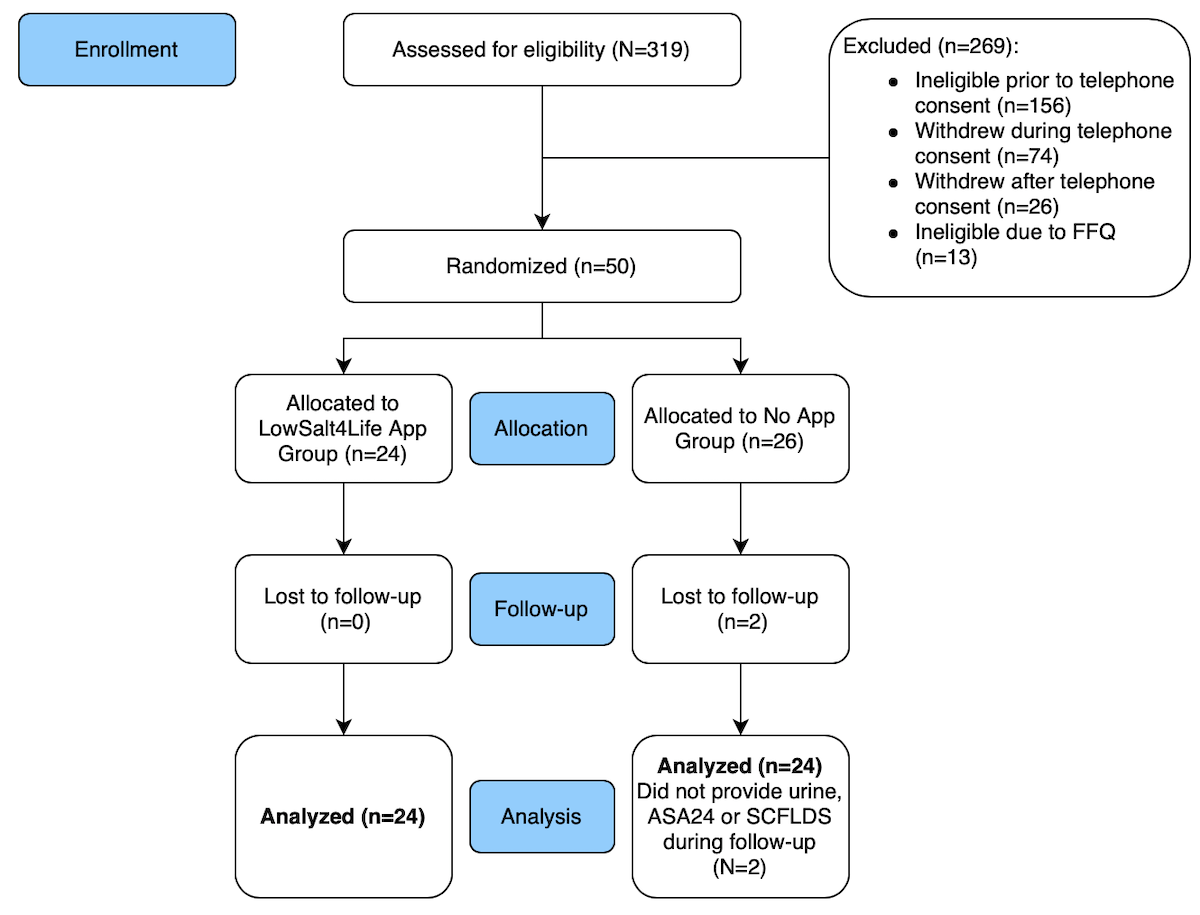
CONSORT flow diagram. ASA24: Automated Self-Administered 24-hour dietary food recall; FFQ: food frequency questionnaire; SCFLDS: Self-care Confidence in Following a Low-sodium Diet Scale.

Fifty patients were enrolled and randomized in the clinical trial (24 in the App group and 26 in the No App group). Figure 3 shows the CONSORT diagram for this clinical trial. There were no significant differences between the 2 groups, except for the SCFLDS. The mean age was 56.6 (SD 10) years in the App group and 58.2 (SD 11) years in the No App group (*P=*.58), and the baseline systolic blood pressure was 129.1 (SD 20) mmHg in the App group and 128.3 (SD 14) mmHg in the No App group. The measures of the sodium intake at baseline were similar between the 2 groups. All baseline measurements are represented in Table 1.

**Table 1:** Baseline demographic data.

Variable		App group (n=24)	No App group (n=26)		*P* value
Age (years), mean (SD)		56.6 (10)	58.2 (11)		.58
Females, n (%)		14 (58)	16 (61)		.82
**Race, n (%)**				.86	
	Caucasian	19 (79)	21 (81)		
	African American	2 (8)	3 (12)		
	Asian	2 (8)	1 (4)		
	Other	1 (4)	1 (4)		
**Ethnicity, n (%)**				>.99	
	Latino	0 (0)	1 (4)		
	Non-Latino	24 (100)	25 (96)		
Previous MI^a^, n (%)		2 (8)	0 (0)		.22
DM^b^, n (%)		0 (0)	1 (4)		>.99
Stroke or TIA^c^, n (%)		0 (0)	1 (4)		>.99
Systolic BP^d^ (mmHg), mean (SD)		129.1 (20)	128.3 (14)		.87
Diastolic BP (mmHg), mean (SD)		84.4 (12)	81 (8)		.23
SCr^e^ (mg/dL), mean (SD)		0.88 (0.1)	0.89 (0.2)		.90
Kawasaki-estimated 24-h urine sodium (mg), mean (SD)		4026 (1514)	3798 (1463)		.59
24-h urine sodium (mg), mean (SD)		3607 (1755)	3561 (1924)		.93
FFQ^f^ sodium (mg/day), mean (SD)		3995 (2119)	3660 (1314)		.51
ASA24^g^ sodium (mg/day), mean (SD)		5127 (3118)	3877 (1773)		.09
Sodium screener (points), mean (SD)		30.3 (11)	31.4 (8)		.70
SCFLDS^h^ (points), mean (SD)		20.9 (5)	18.4 (3)		.04

^a^MI: myocardial infarction.

^b^DM: diabetes mellitus type 2.

^c^TIA: transient ischemic attack.

^d^BP: blood pressure.

^e^SCr: serum creatinine.

^f^FFQ: food frequency questionnaire.

^g^ASA24: Automated Self-Administered 24-hour dietary food recall.

^h^SCFLDS: Self-care Confidence in Following a Low-sodium Diet Scale.

**Figure 4:**
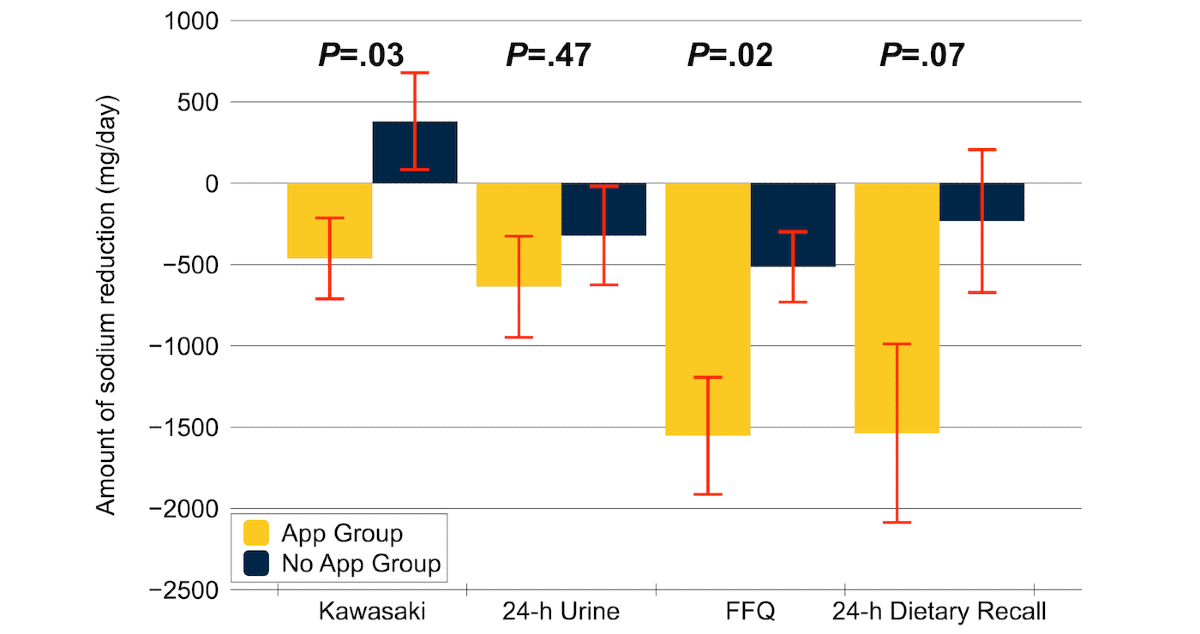
Change in the estimated sodium intake from baseline to 8 weeks. Kawasaki is the 24-h urinary excretion of sodium estimated from spot urine, which was estimated by the Kawasaki equation. This was the primary outcome of the study.

The change in the estimated 24-hour urinary sodium excretion calculated from spot urine from baseline to 8 weeks was –462 (SD 1220) mg in the App group and 381 (SD 1460) mg in the No App group (*P=*.03). The change in the sodium intake measured by the 24-hour urine was –637 (SD 1524) mg in the App group compared to –322 (SD 1485) mg in the No App group (*P=*.47). The change in the estimated sodium intake measured by the ASA24 was –1537 (SD 2693) mg in the App group compared to –233 (SD 2150) mg in the No App group (*P=*.07). The change in the estimated sodium intake by FFQ was –1553 (SD 1764) mg in the App group compared to –515 (SD 1081) mg in the No App group (*P=*.01). Figure 4 shows the change in the estimated sodium intake from baseline to 8 weeks. The sodium intake measurement values at baseline and at 8 weeks are presented in Multimedia Appendix 1. The change in the sodium screen score in the App group was –9.5 (SD 9) points compared to –4.7 (SD 9) points in the No App group over the 8 weeks (*P=*.07). Over time, there was no difference in the change in the self-confidence between the 2 groups (App group 0.08 [SD 5] vs No App group –1.1 [SD 4]; *P=*.38). Analysis of the individual questions did not demonstrate a change in the confidence in reading food labels, shopping at grocery stores, or choosing low-sodium meal options at restaurants.

**Figure 5:**
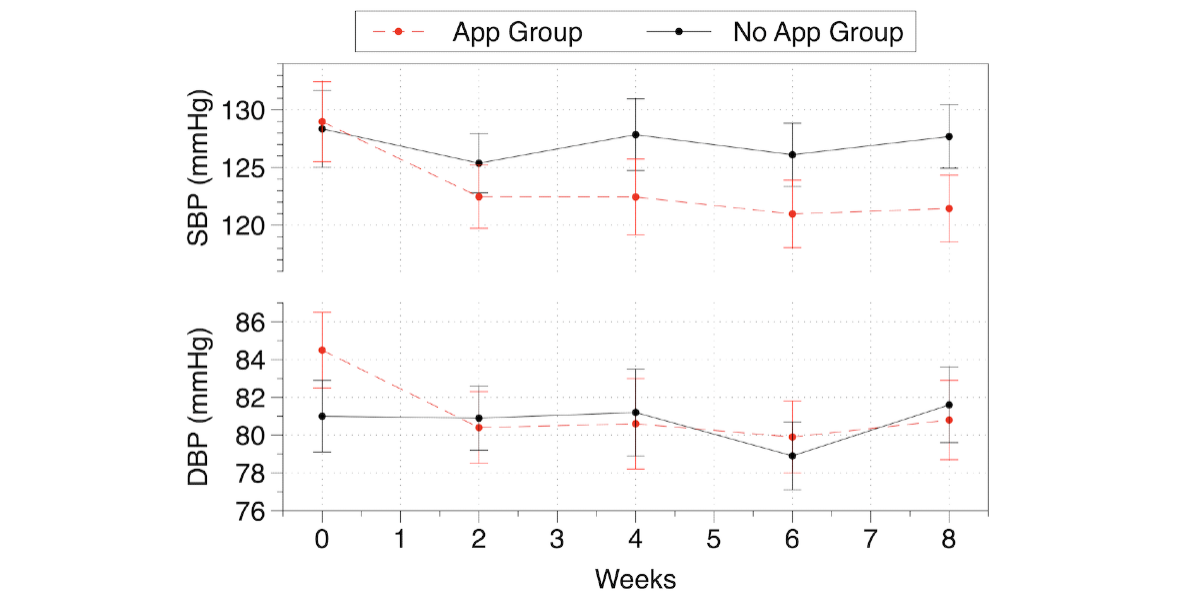
Blood pressure over time. The graph shows mean (SE) generated by least-squared means. DBP: diastolic blood pressure; SBP: systolic blood pressure.

Over the 8 weeks of the study, participants in the App group had 7.5-mmHg reduction in systolic blood pressure (129 mmHg to 121.5 mmHg) compared to a 0.7-mmHg reduction in the No App group (128.4 mmHg to 127.7 mmHg; *P=*.12) based on the least squares means. Figure 5 demonstrates the change in the systolic and diastolic blood pressure levels over time.

**Figure 6:**
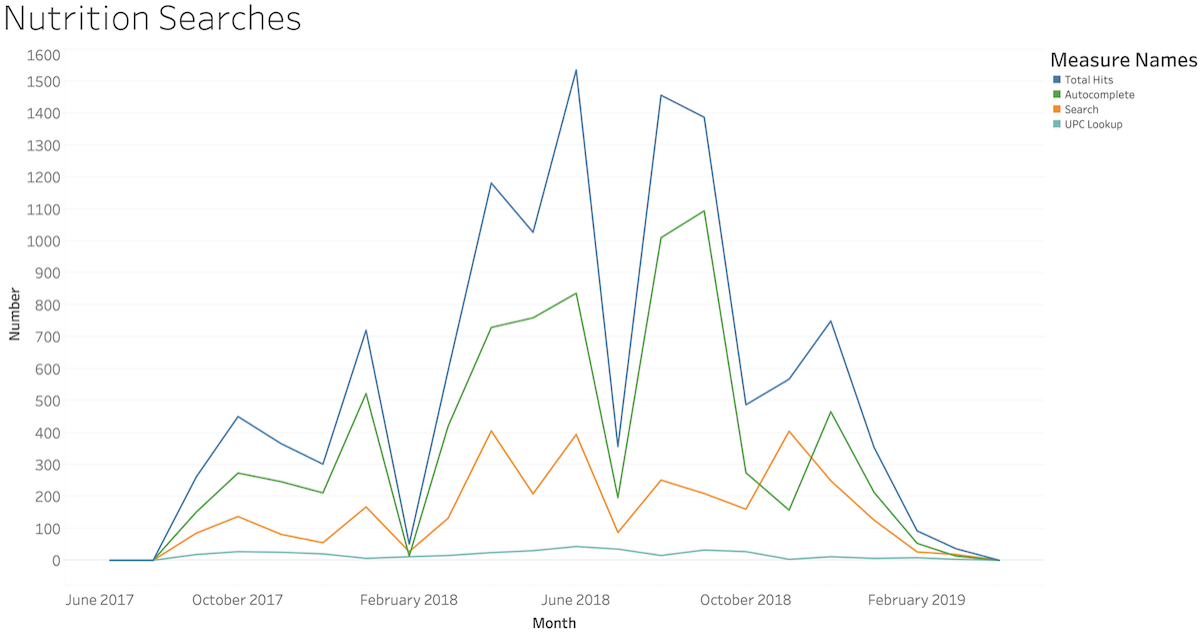
Mobile app usage for nutritional information. UPC: universal product code.

Over the 8 weeks of the study, participants received a median of 126 (IQR 75-186) push notifications: 6 (IQR 3-12) while arriving at the grocery store, 41 (IQR 9-60) while arriving at a restaurant, and 73 (IQR 37-96) while arriving home. Females received a median of 7.5 (IQR 4-15) notifications when arriving at a grocery store compared to 3 (IQR 1-9) notifications received by the males (Wilcoxon *P=*.07). Females received a median of 41 (IQR 9-60) notifications when arriving at a restaurant compared to 34 (IQR 4-61) notifications received by the males (Wilcoxon *P=*.70). There was substantial use of the nutrition information within the mobile app throughout the study. However, the individual participant-level data was not available. The number of overall hits reached 1535 in June 2018. Autocomplete reached 836 searches in June 2018. Universal product code lookup was used 20-40 times per month during the clinical trial. Figure 6 shows the nutrition information searches in the mobile app during the clinical trial. In the survey of the 24 participants who used the app, 19 (79%) agreed that they found the app useful, 19 (79%) agreed that they used the information in the app in their daily life, 17 (71%) agreed that the information they received in the app was important to them, and 20 (83%) agreed the app was easy to use. Only 3 (13%) participants found the app confusing and 1 (4%) found the app difficult to understand.

## Discussion

In this pilot study, we found that LowSalt4Life, a just-in-time adaptive mobile app intervention that recommends lower dietary sodium food alternatives at home, restaurants, and grocery stores, holds promise in reducing the dietary sodium intake and systolic blood pressure. We demonstrated that compared to the control, the intervention group had a greater reduction in the dietary sodium intake over 8 weeks, as measured by the estimated 24-hour urinary sodium excretion from spot urine and FFQ. However, we did not observe a statistically significant difference between the 2 groups with regard to the self-confidence of the individuals in following a low-sodium diet, 24-hour urinary sodium excretion, dietary intake of sodium measured by 24-hour dietary recall or systolic blood pressure over 8 weeks. Although some of these measurements were not statistically significant, the measures of dietary sodium intake and systolic blood pressure showed clinically significant improvements in the intervention group compared to those in the control group. We believe the mechanism of the intervention is identifying high-sodium foods and providing lower sodium alternatives to those foods at the time of eating or purchasing the food. In a recent study on consumer understanding of sodium intake and food labeling, only half of the grocery store customers were able to correctly use the sodium label information to choose low-sodium foods [19]. The American Heart Association guideline for the dietary approach to prevent and treat hypertension states that “any meaningful strategy to reduce salt intake must involve the efforts of food manufacturers and restaurants” [20].

We are currently witnessing broad social changes in how individuals expect to find and use information about their health. According to the Pew Research Center’s Internet and American Life Project, 73% of the households have broadband service and 81% of the Americans (53% of them >65 years) have a smartphone [21], which supports the need for mobile app–based interventions for health care. In a recent systematic review of mobile health interventions to lower sodium intake, only 6 were randomized controlled clinical trials and only 2 of those trials were mobile app interventions published in English [22]. SaltSwitch (New Zealand) is a mobile app focused on supporting users to find food options with low sodium levels at grocery stores [23]. SaltSwitch provides low-sodium alternatives for items scanned at grocery stores but is not personalized for the user’s requirements of high-sodium foods; moreover, it does not provide a push notification on entry to remind the user to scan the items and does not provide contextual food information at restaurants. The 4-week randomized controlled trial in 66 patients with cardiovascular disease demonstrated that those randomized to the SaltSwitch intervention group purchased foods with low sodium levels more often when compared to the control, but the intervention did not reduce urinary sodium excretion or blood pressure over time. Another study randomized 30 adults to the MyFitnessPal app for food logging to receive sodium content feedback or a paper journal of food logging for sodium content feedback for 4 weeks [24]. Although the MyFitnessPal app provided personalized dietary information about the sodium content of the foods, specific recommendations on what food substitutions could be done at different locations were lacking. The change in the predicted urinary sodium excretion from a spot urine test over 4 weeks was significantly greater in the MyFitnessPal group (–838 [SD 1093] mg) compared to that in the paper journal group (236 [SD 1333] mg). These mobile apps, in addition to our study app, show that mobile app–based interventions can improve patient health behaviors over a short time. The major difference between LowSalt4Life and these interventions is the just-in-time adaptive intervention that we deployed. The just-in-time adaptive intervention is a novel approach that makes mobile interventions “smarter” by incorporating real-time data streams to generate tailored notifications and then deliver these notifications at key moments when there is a high likelihood of success. Just-in-time adaptive interventions have shown efficacy in multiple treatment domains, including smoking cessation, alcohol abuse, and mental health treatment [25,26].

Interestingly, the participants’ confidence in following a low-sodium diet was different at baseline and was not affected by the intervention. There was no difference between the groups over time for specific questions from the survey about the grocery stores and restaurants. This supports the theory that the intervention does not affect patient self-confidence; in fact, the participants in our study were somewhat confident to very confident in following a low-sodium diet at baseline. Improvement in confidence may have been difficult, given such high confidence at the baseline. However, this confidence did not appear to be warranted, illustrating the unmet need for additional assistance with an intervention. It could also be that the participants require a longer duration of the intervention to feel more self-confident. Our intervention period was relatively short—only 8 weeks.

Our study had the following limitations. First, there were baseline differences in the confidence in following a low-sodium diet. It is unclear if this difference in confidence is clinically significant, but nevertheless, future research studies should investigate this finding further. Second, several of the sodium intake measures did not demonstrate statistically significant changes. However, all the measures of sodium intake present methodological challenges [27], and the trend toward improvement in the App group was consistent. Subjective measurements such as FFQ and 24-hour dietary recall have recall bias. Urinary measurements of sodium intake have high levels of random errors due to day-to-day variations. Third, all participants were required to have an iPhone, and enrollment was performed at 1 institution. This could have led to skewed socioeconomic backgrounds in our study compared to the general population diagnosed with hypertension. Fourth, the clinical trial was set up as an App versus No App study wherein the control group did not receive the app. This could have led to a lack of an attention control group. Participants could have felt left out of the intervention arm and not engaged enough in the study. However, the No App group participants did consistently participate by providing blood pressure measurements, dietary survey responses, and urinary collections over the 8-week study. To minimize potential bias, future studies could be designed with an attention control, sequential multiple assignment randomization, or microrandomization in the control group.

In conclusion, our randomized controlled 8-week pilot study in adults with hypertension showed that a contextual just-in-time mobile app intervention resulted in a greater reduction in dietary sodium intake as measured by the estimated 24-hour urinary sodium excretion from spot urine and FFQ compared to that in the control. The intervention group did not show statistically significant differences from the control in the self-confidence in following a low-sodium diet, 24-hour urinary sodium excretion, or dietary intake of sodium as measured by the 24-hour dietary recall over 8 weeks. A larger clinical trial is warranted to further elucidate the effects of the LowSalt4Life intervention on sodium intake and blood pressure.

## Appendix

Multimedia Appendix 1 Estimates of dietary sodium intake at baseline and week 8.

## Abbreviations

API: application programming interface
ASA24: Automated Self-Administered 24-hour dietary recall
CKD: chronic kidney disease
FFQ: food frequency questionnaire
SCFLDS: Self-care Confidence in Following a Low-sodium Diet Scale
