# Supplementary material for: Effects of a Novel Contextual Just-In-Time Mobile App Intervention (LowSalt4Life) on Sodium Intake in Adults With Hypertension: Pilot Randomized Controlled Trial
Source: JMIR Mhealth Uhealth. 2020 Aug 10;8(8):e16696. doi: 10.2196/16696 (PMC7445610; doi:10.2196/16696)
Supplement: Multimedia Appendix 1 [file mhealth_v8i8e16696_app1.docx]

**Appendix 1: Estimates of dietary sodium intake at baseline and week 8.**

| **Variable** | **App** | | **No App** | |
| --- | --- | --- | --- | --- |
|  | **Baseline** | **Week 8** | **Baseline** | **Week 8** |
| Kawasaki estimated 24-hr urinary excretion of sodium, mg | 4026.1 ± 1514 | 3563.9 ±1121 | 3797.5 ± 1463 | 4201.3 ± 1594 |
| 24-hr urinary excretion of sodium, mg | 3606.5 ± 1755 | 2970 ± 1475 | 3560.6 ± 1924 | 3411.8 ± 1368 |
| ASA24, mg/day | 5126.9 ± 3118 | 3589.7 ± 1820 | 3877.2 ± 1773 | 3695.1 ± 1681 |
| FFQ, mg/day | 3994.9 ± 2119 | 2441 ± 1132 | 3660.4 ± 1314 | 3156.3 ± 1147 |
